# A baseline profile of the Queensland Cardiac Record Linkage Cohort (QCard) study

**DOI:** 10.1186/s12872-022-02478-z

**Published:** 2022-02-05

**Authors:** Son Nghiem, Clifford Afoakwah, Paul Scuffham, Joshua Byrnes

**Affiliations:** 1 Centre for Applied Health Economics, Griffith University, 117 Kessels Road, Nathan, Brisbane, QLD 4111 Australia; 2 Menzies Health Institute Queensland, Griffith University, Level 8.86, G40-Griffith Health Centre, Gold Coast, QLD 4222 Australia

**Keywords:** Cardiovascular diseases, Record linkage cohort study, Queensland, Australia, Baseline profile

## Abstract

**Background:**

Cardiovascular disease (CVD) is one of the leading causes of death in Australia. Longitudinal record linkage studies have the potency to influence clinical decision making to improve cardiac health. This paper describes the baseline characteristics of the Queensland Cardiac Record Linkage Cohort study (QCard).

**Methods:**

International Classification of Disease, 10th Revision Australian Modification (ICD-10-AM) diagnosis codes were used to identify CVD and comorbidities. Cost and adverse health outcomes (e.g., comorbidities, hospital-acquired complications) were compared between first-time and recurrent admissions. Descriptive statistics and standard tests were used to analyse the baseline data.

**Results:**

There were 132,343 patients with hospitalisations in 2010, of which 47% were recurrent admissions, and 53% were males. There were systematic differences between characteristics of recurrent and first-time hospitalisations. Patients with recurrent episodes were nine years older (70 vs. 61; p < 0.001) and experienced a twice higher risk of multiple comorbidities (3.17 vs. 1.59; p < 0.001). CVD index hospitalisations were concentrated in large metropolitan hospitals.

**Conclusions:**

Our study demonstrates that linked administrative health data provide an effective tool to investigate factors determining the progress of heart disease. Our main finding suggests that recurrent admissions were associated with higher hospital costs and a higher risk of having adverse outcomes.

**Supplementary Information:**

The online version contains supplementary material available at 10.1186/s12872-022-02478-z.

## Background

Cardiovascular disease (CVD) is the leading cause of death and injury globally and Australia [1]. It is estimated that 32% of all-cause death in 2019 were attributable to CVD, with heart attack and stroke accounting for 85% [2]. In Australia, one in every four deaths was CVD-related in 2018 [3]. The cost of CVD has increased rapidly, accounting for 12% of total health expenditure [4]. Queensland is Australia hotspot for CVD, with the incidence of acute coronary events (e.g., heart attacks) were 20% higher than the national average in 2014. One-tenth of all hospital expenditure was related to CVD, and the CVD death rate was 5% higher than the national rate in 2014 [3]. Despite the severity and the high burden of CVD in Australia, inequality in access to healthcare, and the attainment of health outcomes, remain an issue among cardiac patients.

A record-linkage cohort study is one of the effective ways to understand better the nature and extent of inequality in cardiac healthcare use and outcomes. Such information is important for adequate and reliable delivery of public health services and secondary prevention to avoid rehospitalisation and to maintain the best possible health for people with CVD [5, 6]. This study describes the baseline characteristics of the Queensland Cardiac Record Linkage Cohort (QCard) [7] study that included 135,399 cardiac patients with six years follow-ups across 247 health facilities.

This paper presents comprehensive baseline characteristics of index CVD hospitalisations (i.e., the first-time CVD hospital admission of QCard participants) as well as a comparative analysis between first-time and recurrent admissions. This baseline profile provides a reference point for future research on various issues such as disease progression and determinants of health outcomes.

## Methods

### Study design

QCard is a record linkage longitudinal cohort of CVD patients in Queensland, Australia. The cohort includes all CVD hospitalisations indexed in 2010 and followed until the end of 2015. The main data set of this study was Queensland Hospital Admitted Patient Data Collection (QHAPDC). This data set was linked, using a probabilistic linkage method [8], with various other administrative health data sets, including Emergency Department Data Collection (EDDC), Registrar General Deaths Database (RGDD), sub-acute and non-acute admission, comorbidity diagnosis, hospital costs, the Medicare Benefits Schedule (MBS) and the Pharmaceutical Benefits Scheme (PBS). The cohort was also linked with various external data sets, including environmental pollution and macroeconomic indicators. A deterministic linkage method [9] was selected to link external data because aggregated linkage fields (e.g., residential postcodes), which were better to protect the privacy of patients. Detailed data linkage process and description of variables are presented in the cohort protocol [7]. All admission records were deidentified before the linkage process.

### Variable descriptions

The cohort includes information about patients, admission episodes, hospitals, and residential areas. Patients’ characteristics include age, sex, ethnicity, marital status, health insurance status, regions of origin, and residential areas (e.g., postcodes). The episode characteristics include whether the patient was admitted via the emergency department (ED), ED discharge status, sources of referral, types of admission (same day, overnight, and multiple days), whether the admission was acute, whether the episode was admitted at a private health facility (the mixed health care system in Australia allow public patients admitted at private hospitals or private patients at admitted at public hospitals [10]), wards types (e.g., private-single, private-shared, and public wards), condition onset flag (i.e., whether the condition appeared upon arrival or during admission) and International Classification of Diseases, 10th Revision (ICD-10) diagnosis codes. Based on diagnosis codes, we estimated other health outcomes, including the Charlson comorbidity index [11] and hospital-acquired complications (HAC) [12]. The episode characteristics also include hospital costs, MBS and PBS records of health services and medication consumption. Finally, characteristics of residential areas, which were linked via postcodes, were included in the cohort. In this baseline study, we only present the socio-economic index for areas (SEIFA) [13], which is a composite index constructed from various variables, including income, education, housing and employment. The index was standardized to a score of 0 to 1000, where higher score indicates a higher socio-economic advantage of the region. In this baseline study, we use the quintiles of SEIFA to represent a socio-economic advantage. The residents of postcodes with the highest SEIFA quintile (SEIFA-Q5) are assumed to have the highest socioeconomic advantage.

### Study population

The study population includes 1.8 million CVD admissions of 135,399 patients who were hospitalised in Queensland in 2010 and followed up until 2015. The inclusion criteria for this study was any patients aged 18 and above who was hospitalised in 2010 for any CVD-related condition. CVDs were defined as having the first three characters, or the section heading, of ICD-10 diagnosis codes in the range of I00-I99 [14].

Patients with an admission date before 2010 were excluded. Most patients in this group were retrieved in the original data set because their discharged date was in 2010 (e.g., patients admitted in December 2009 and discharged in January 2010). However, the original data set also included some patients admitted long before 2010, which could be due to retrieval errors.

### Analysis

Baseline analyses were conducted using summary statistics, trends, patterns and correlations. Statistical tests, including t-test [15] and Kruskal–Wallis test [16], were used respectively to explore differences in means and medians characteristics between first-time and recurrence admissions. Spatial information (e.g., boundary shapes, longitudes, and latitudes) were used to illustrate the variations in access to healthcare and selected health outcomes by geographical locations.

ICD-10 codes were used to estimate various outcomes of interest using relevant algorithms. In particular, the HAC were identified using the latest algorithm (HAC Version 3.0) developed by the Australian Commission in Safety and Quality in Health Care [12, 17]. In this study, we focus on presenting HAC-14 − cardiac HACs. A cardiac HAC was defined as having at least one subsequent diagnosis code (i.e., primary diagnosis codes were excluded) that occurred during hospitalisation (i.e., not presented upon admission and not recorded as a principal diagnosis) in one of the five groups. The five groups include (1) Heart failure and pulmonary oedema (ICD-10 codes: I50.[0,1,9]); (2) arrhythmias (I47.[0,1], I49.[0,8,9], R00.1); (3) cardiac arrest (I46.[0,1,9]); (4) acute coronary syndrome (I120.0, I121.[0,1/4,9], I122.[0,1,8,9]); and (5) infective endocarditis (I33.0).

We also used ICD-10 diagnoses codes to calculate the updated Charlson comorbidity index [11]. The updated Charlson comorbidities index was constructed from 12 comorbidities that were mostly correlated with the risk of one-year mortality. Only secondary diagnosis codes with an onset flag of “present on admission” were used to calculate the comorbidity index. The index was validated using data from six countries (Australia, Canada, New Zealand, France, Japan, Switzerland) with excellent discrimination power in predicting mortality. Details of the algorithm to calculate the Charlson comorbidity index was presented in Quan et al. [18]. Data were analysed using Stata 15 [19] and R 3.6.1 [20].

## Results

### Admission types

**Fig. 1:**
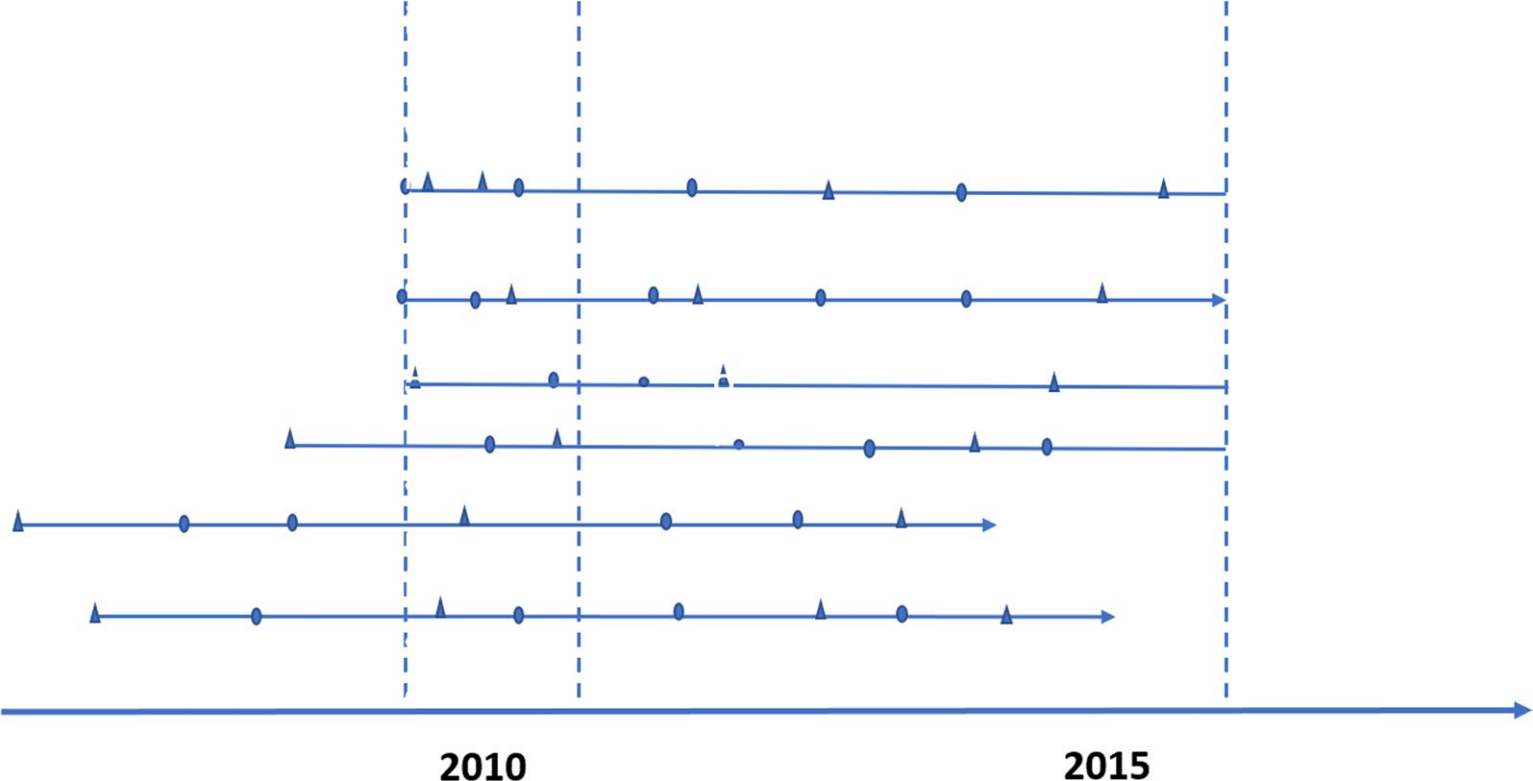
Timeline and classification of admissions. Circles represent admissions with CVD identified in a subsequent diagnosis; triangles represent admissions with CVD identified in the principal diagnosis. Index admissions were the first admissions that occurred in 2010. Incident admissions refer to admissions that index admissions coincided with the first-time admissions (first three lines). Recurrent admissions refer to admissions where the first-time admissions occurred before 2010 (last three lines)

After removing duplications, retrieval errors, and mismatching records from the original 135,399 patients, the population of the baseline study included 132,343 index hospitalisations across 247 hospitals. These hospitalisations also represent 132,343 patients because each patient had only one index hospitalisation (see Fig. 1, where index hospitalisations were represented as triangle shapes). Note that index hospitalisations may not necessarily be the first hospitalisations because the cohort also includes admissions with CVDs identified only in subsequent diagnosis codes, represented as circle shapes in Fig. 1. Although detailed admissions were only observed in 2010–2015, the cohort includes information on the date of first-ever CVD admission. Some patients in this cohort have the first CVD admission occur before 2010, hereafter referred to as recurrent episodes since their index hospitalisation in 2010 were not the first-ever CVD admissions (i.e., the bottom three lines in Fig. 1). The remaining hospitalisations were referred to as first-time admissions (i.e., the top three lines in Fig. 1) because those index hospitalisations were also their first-ever CVD admissions.

### Descriptive characteristics

Table 1 shows that out of the 132,343 patients with index hospitalisations in 2010, there were 56,514 patients (42.7%) who had a first CVD admission that occurred before 2010 (i.e., recurrent admissions). The remaining 75,829 index hospitalisation episodes were first CVD admissions that occurred after 2010 (i.e., first-time admissions). The average age of hospitalisations was 65 years, with patients of recurrent admissions were older (71 years) than the first-time admissions (62 years). Also, males dominate CVD admissions with 53%, while indigenous patients accounted for only 3% of all cardiac admissions in 2010, which is similar to the average indigenous population in Australia [21].

**Table 1:** Baseline characteristics at index admissions

Variables	First-time admissions (N = 75,829)	Recurrent admissions (N = 56,514)	All (N = 132,343)	p-value
Age	61.62 (16.81)	70.7 (14.17)	65.5 (16.37)	< .001
Charlson index	1.59 (2.56)	3.17 (3.09)	2.28 (2.91)	< .001
Sex (male = 1)	39,522 (0.52)	30,611 (0.54)	70,133 (0.53)	< .001
Indigenous (yes = 1)	1407 (0.02)	1952 (0.03)	3359 (0.03)	< .001
Hospital insurance (yes = 1)	39,779 (0.52)	23,968 (0.42)	63,747 (0.48)	< .001
Private hospitals (yes = 1)	37,806 (0.50)	20,771 (0.37)	58,577 (0.44)	< .001
Ward: Private shared	27,275 (0.36)	14,722 (0.26)	41,997 (0.32)	< .001
Ward: Private single	12,658 (0.17)	8240 (0.15)	20,898 (0.16)	< .001
Ward: Public	35,896 (0.47)	33,552 (0.59)	69,448 (0.52)	< .001
SEIFA Q1	12,282 (0.16)	10,938 (0.19)	23,220 (0.18)	< .001
SEIFA Q2	13,415 (0.18)	10,942 (0.19)	24,357 (0.18)	< .001
SEIFA Q3	15,222 (0.20)	11,948 (0.21)	27,170 (0.21)	< .001
SEIFA Q4	16,358 (0.22)	11,049 (0.20)	27,407 (0.21)	< .001
SEIFA Q5	17,982 (0.24)	11,568 (0.20)	29,550 (0.22)	< .001
Married	48,475 (0.64)	32,313 (0.57)	80,788 (0.61)	< .001
Widowed	9258 (0.12)	12,231 (0.22)	21,489 (0.16)	< .001
Never married	10,950 (0.14)	5864 (0.10)	16,814 (0.13)	< .001
Divorced separated	7146 (0.09)	6106 (0.11)	13,252 (0.10)	< .001
Multiday	35,664 (0.47)	34,942 (0.62)	70,606 (0.53)	< .001
Overnight	12,562 (0.17)	9227 (0.16)	21,789 (0.16)	< .001
Same-day	27,603 (0.36)	12,345 (0.22)	39,948 (0.30)	0.260
ED usage	39,574 (0.52)	37,404 (0.66)	76,978 (0.58)	< .001
Referrals: ED	31,534 (0.42)	30,008 (0.53)	61,542 (0.47)	< .001
Referrals: Private facilities	31,378 (0.41)	15,379 (0.27)	46,757 (0.35)	< .001
Referrals: Outpatients	8968 (0.12)	7657 (0.14)	16,625 (0.13)	< .001
Referrals: Transfer	2383 (0.03)	1613 (0.03)	3996 (0.03)	< .001
Comorbidity: 0	39,634 (0.55)	13,352 (0.24)	52,986 (0.41)	< .001
Comorbidity: 1	9414 (0.13)	7996 (0.14)	17,410 (0.14)	< .001
Comorbidity: 2+	23,592 (0.33)	34,533 (0.62)	58,125 (0.45)	< .001
Cardiac HAC	2562 (0.03)	2260 (0.04)	4822 (0.04)	< .001
Multiple day Cardiac HAC	2352 (0.07)	2105 (0.06)	4457 (0.06)	< .001
Acute admissions	75,012 (0.99)	55,455 (0.98)	130,467 (0.99)	< .001

Baseline characteristics are presented as means (standard deviation) in the first two rows and frequency (percent) in the remaining. P-values represent t-test and Kruskal–Wallis test, respectively, differences in mean and median by incidence status of continuous and binary variables. We also conduct a Chi-squared test for categorical variables (e.g., SEIFA quintiles) and found that p-values were all < 0.01

Table 1 further shows that 48% of all hospitalised patients had private health insurance. This is reflected in the use of private hospitals as about 37% of recurrence hospitalisations and 50% of first-time hospitalisations utilised private health facilities. The rate of admission to private wards in public hospitals was also higher for first-time hospitalisations (53%) compared to recurrence hospitalisations (41%). SEIFA quintiles were evenly distributed among recurrence admissions, while higher SEIFA quintiles were slightly over-represented among first-time admissions. At index hospitalisations, 61% of patients were married, but the second most popular marital status differ considerably between the two admission groups. For recurrence admissions, widowed was the next popular group with 22%, while “never married” was the next popular marital status for first-time admissions with 14%.

Regarding admission characteristics, same-day admissions accounted for 30%, and the rate is not statistically different between recurrence and first-time groups. In contrast, multiple-day admission rates were substantially higher among recurrence hospitalisations (62%) than that of first-time hospitalisations (47%). Most index hospitalisations were admitted from the ED services, and the ED usage rate was higher among recurrence episodes (66%) than that of first-time episodes (52%). EDs were the most common source of referral with 47% of all index hospitalisations, although this rate is lower for first-time episodes, with 42% compared to 53% for recurrence episodes. A reverse trend occurs in the second most common source of referrals—private facilities—with 41% for first-time hospitalisations and 27% for recurrence hospitalisations.

### Selected outcomes

The Charlson comorbidity index showed that recurrence episodes experienced almost twice higher in comorbidity score (3.17) compared to that of first-time episodes (1.59). The comorbidity classification also reveals a similar finding: 45% of index hospitalisations have at least two comorbidities, and there was a sharp contrast with 62% in recurrence episodes and 35% in first-time episodes. The largest proportion (53%) of first-time episodes have no comorbidity. The rate of CVD-related HAC was 4%, and most HACs occurred among multiple-day episodes. One surprising observation is that the rate of CVD-related multiple-day HAC was slightly higher among first-time episodes, which could be due to the relatively lower rate of multiple-day admission among first-time episodes (47% vs. 62%). The majority (99%) of index hospitalisations were due to sudden illness (acute episodes) despite the rate was lower among recurrence episodes, suggesting that sustain illness (i.e., chronic episodes) was higher in this group. Overall, the baseline characteristics of index hospitalisations show a systematic difference between recurrence and first-time episodes.

### Hospital characteristics

**Fig. 2:**
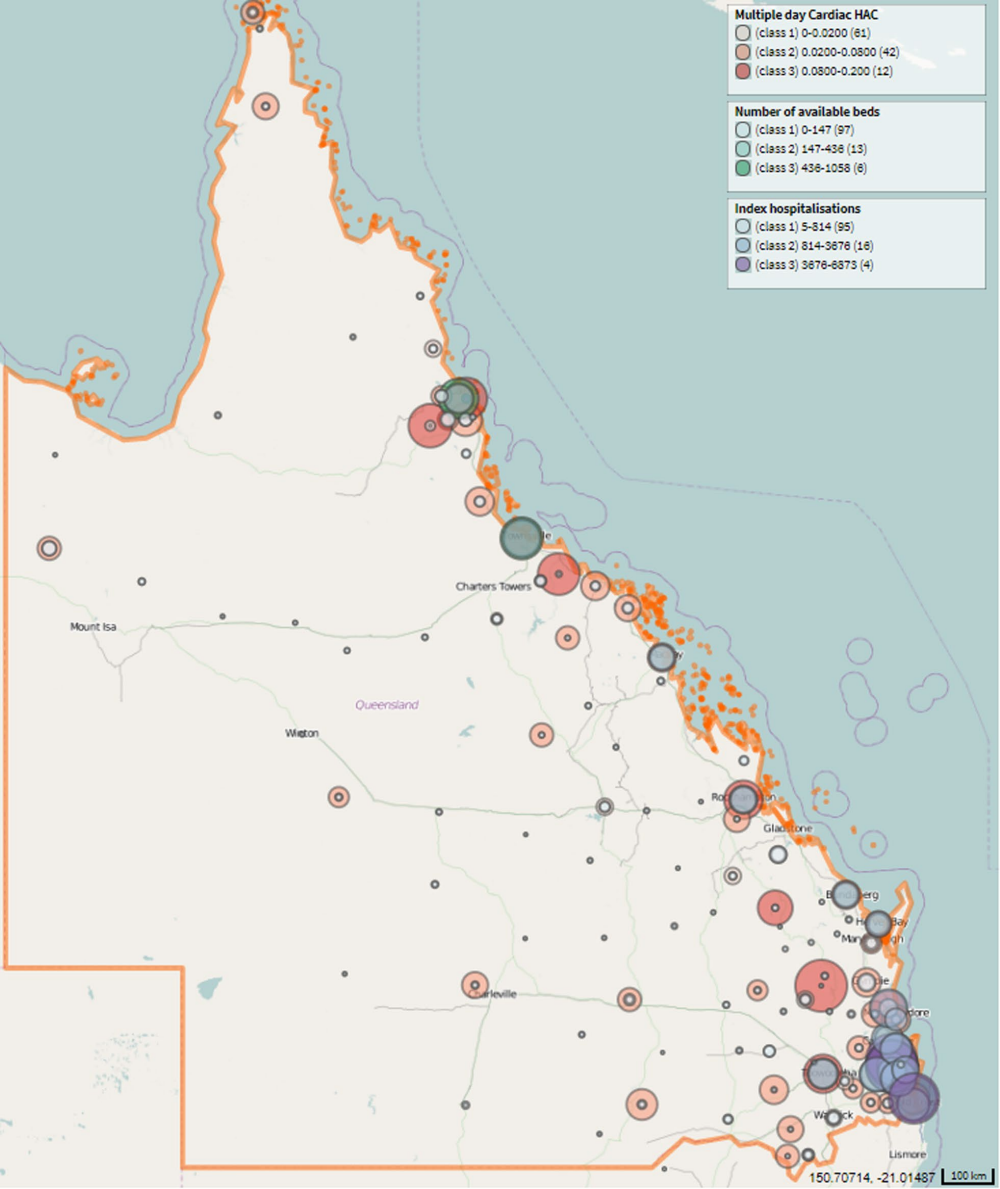
Spatial distributions of index admissions and HAC by hospitals. Own illustration by the authors

The distribution of first-time episodes shows that large metropolitan hospitals account for a larger share of admissions. Also, hospitals with a larger number of beds available, which were located mostly in metropolitan areas, received a higher frequency of index hospitalisations. The rate of multiple-day cardiac HAC also varied substantially between hospitals, ranging from 0 to 20%. In addition, hospitals with higher HAC rates were located in both regional areas and metropolitans. The concentration of admissions in major cities suggests that people in outback regions could face adverse health outcomes due to the vast distance to metropolitan hospitals (Fig. 2).

### Frequent diagnoses

The top 10 ICD-10 codes showed hypertension (high blood pressure) was the most common condition, but recurrence patients experience a higher rate with 40%, while the respective figure for first-time episodes was 27% (see Supplement material for details). The remaining conditions in the top 10 differ considerably between the two groups. For recurrence admissions, the next two common conditions are atrial fibrillation and chronic ischemic heart disease. In contrast, the respective conditions for first-time admissions were internal thrombosed haemorrhoids and chronic ischemic heart disease. The high prevalence rate of internal thrombosed haemorrhoids, which is a vascular disease [22], in CVD admissions seem surprising, but this condition was often associated with poor circulation of blood from veins to the heart, which in turn, resulting from a blood clot or impaired blood circulation in lower rectum veins [23, 24]. Heart failure is the next most common condition for recurrence episodes, but for first-time episodes, it was hypotension (low blood pressure). The order of the remaining conditions was similar for both groups: angina, myocardial infarction (heart attack), atherosclerosis (obstruction of arteries by the decomposition of fatty materials), and paroxysmal tachycardia.

### Health services consumption

The consumption of health services and medications within 30 days before the index hospitalisations show a similar rate between the two groups (see Supplementary materials). For example, the most common types of health services consumed in 30 days before the index hospitalisation were general practice (GP) attendance with 67% of patients attended, followed by attendance to specialists (48%) and consultant physicians (46%). Specialist attendance is the only health service that the usage rate is not statistically different between recurrence and first-time episodes. Surgical-related health services (e.g., Anaesthetics and surgical operations) and diagnoses services (e.g., ultrasound, diagnosis radiology) were also commonly used within one month before index hospitalisation.

Half of the top 10 medications used within 30 days prior to index hospitalisations, according to the Anatomical Therapeutic Chemical (ATC) codes, were mostly Angiotensin-converting enzyme (ACE) inhibitors, beta-blockers and lipid modifiers. These medications are commonly used for CVD-related conditions such as high blood pressure, heart failure and angina. This observation suggests that patients in this cohort were diagnosed with CVD-related conditions before their first hospitalisations. However, the rate of medication usage was substantially lower among first-time episodes.

## Discussion

The baseline characterises of the QCard cohort revealed that CVDs occur mostly in older patients with an average age of 65 years for first-time admissions. Also, males were slightly over-represented, with 53% of first-time episodes. These observations were in line with the literature that older people and males faced a higher risk of CVD [25]. Widowers are the second-most popular group after married, and the share of widowers was lower among first-time episodes. This observation reflects the fact that CVDs incurred mostly among older adults and females have a longer life expectancy than males [26]. The positive correlation between CVD incidence and age may also explain the relatively higher CVD incidence among people from high socio-economic groups because they tend to live longer [27].

One interesting observation from the baseline characteristics is that more than half of the patients were covered by private health insurance, although Australia has a universal health care system. Incentives such as tax rebates and age-adjusted premiums [28] have resulted in increased private health insurance coverage and hence increased the choice of private care [29]. Although this cohort includes mostly public hospitals, patients with private health insurance coverage enjoyed benefits such as private wards or shorter waiting times for elective care. Public patients with private health insurance coverage may also prefer admission to private facilities.

The spatial distribution of CVD index hospitalisations and health outcomes also suggested that people in remote communities may face challenges in access to CVD care on time. Apart from the vast transfer distance, the difference in operational scale between small regional hospitals and large to metropolitan hospitals may explain the higher rates of HAC in both types of facilities. For example, larger hospitals were better equipped to treat complex CVD conditions (i.e., sicker patients), which may result in higher HAC rates. In contrast, the lack of equipment and/or skilled staff in remote facilities may explain their high HAC rates.

The comparison of health outcomes between the first-time and recurrence hospitalisations in this study rejected the null hypothesis of no systematic difference. This sets up a solid foundation for the next analysis to model the progression of CVD conditions. Lessons learnt from this QCard can be translated easily to model the progression of other diseases using record linkage of admission data. Also, results of disease progression models (e.g., transition probability matrix) provide crucial inputs to estimate the cost-effectiveness of health interventions in practices. Differences (if any) between cost-effectiveness results at the trial stage and the implementation stage can provide policy applications for a more cost-effective health care system in practice.

Although CVD cohort linkage studies have been conducted previously [30–33], including Australia [34–37], most of the studies have focused on specific types of CVD, such as myocardial infarction [30], heart failure [31, 34–36], acute coronary syndrome [32], coronary heart disease [37], and obstructive coronary artery disease [33]. This linkage cohort study covers all types of CVDs, which enable studies of both specific diseases and interactions among diseases. The ability to investigate interactions between different CVD diseases is crucial because health and CVDs are complex with many comorbidities; hence, singling out one specific condition only reveals a partial picture of CVDs. In addition, most previous studies have focused on randomized control trials (RCTs), which represent the gold standard in finding clinical effects. However, RCTs often contain relatively small sample sizes, and hence, have weak external validity. Also, not all results from RTCs are translated into practices.

That said, the strength of this study is the use of large-scale linked administrative data, which allows us to follow patients over six years to examine the progression of CVD in the population. Another strength of this cohort is its ability to be linked with a wide range of related data sets. Thus, the cohort provides comprehensive and robust information to investigate the burden of diseases, socio-economic determinants of health as well as the impact of health policies on outcomes.

Despite the outlined novelty of this study, some limitations cannot be escaped. First, it follows only those with a CVD hospitalisation in 2010 and does not include other patients with a CVD hospitalisation after this census year. Thus, the cohort is not suitable to examine the new development of the disease (e.g., the incidence of CVD in 2011 onwards). Second, a follow-up period of six years may be too short to estimate the disease progression and survival profile of most patients. Hence, follow-up data beyond 2015 will provide useful information on disease progression and healthcare use among cardiac patients.

## Conclusions

This study has described baseline the characteristics o index hospitalisations of the QCard. The baseline characteristics are in line with the literature that older adults and males are more at risk of CVD. The QCard cohort provides rich information for further investigation of CVD disease progression and its determinants. Experience from this study also provides useful insights for the expansion of this study to other states in Australia or other countries and broader clinical areas.

## Supplementary Information

**Additional file 1.** Appendices.

## Data Availability

The data that support the findings of this study are available from Queensland Health, but restrictions apply to the availability of these data, which were used under license for the current study, and so are not publicly available. Data are, however, available from the authors upon reasonable request and with permission of Queensland Health.

## Abbreviations

ACE: Angiotensin-converting enzyme
ATC: Anatomical therapeutic chemical
CVD: Cardiovascular disease
ED: Emergency Department
EDDC: Emergency Department Data Collection
GP: General practice
HAC: Hospital-acquired complication
ICD-10: International Classification of Diseases, 10th Revision
MBS: Medicare Benefits Schedule
PBS: Pharmaceutical Benefits Scheme
QCARD: Queensland Cardiac Record Linkage Cohort
QHAPDC: Queensland Hospital Admitted Patient Data Collection
RGDD: Registrar General Deaths Database
SEIFA: Socio-economic index for areas
