# Supplementary material for: A baseline profile of the Queensland Cardiac Record Linkage Cohort (QCard) study
Source: BMC Cardiovasc Disord. 2022 Feb 5;22:35. doi: 10.1186/s12872-022-02478-z (PMC8817516; doi:10.1186/s12872-022-02478-z)
Supplement: Supplementary file 1 — Additional file 1. Appendices. [file 12872_2022_2478_MOESM1_ESM.docx]

**Table S1.** Top 10 CVD conditions at index hospitalisations

| ICD-10 codes | Descriptions | Frequency (rate) | | |
| --- | --- | --- | --- | --- |
|  |  | Recurrence | Incidence | All |
| I10 | Hypertension | 22827  (0.40) | 20395 (0.27) | 43222 (0.33) |
| I84 | Internal Thrombosed Haemorrhoids | 5274  (0.09) | 19954 (0.26) | 25228 (0.19) |
| I25 | Chronic ischemic heart disease | 10833  (0.19) | 9259 (0.12) | 20092 (0.15) |
| I48 | Atrial fibrillation | 10850  (0.19) | 8793 (0.12) | 19643 (0.15) |
| I95 | Hypotension | 5895  (0.1) | 7452 (0.1) | 13347 (0.1) |
| I50 | Heart failure | 7353  (0.13) | 3928 (0.05) | 11281 (0.09) |
| I20 | Angina | 5828  (0.1) | 5296 (0.07) | 11124 (0.08) |
| I21 | Myocardial infarction | 3281  (0.06) | 5218 (0.07) | 8499 (0.06) |
| I70 | Atherosclerosis | 2643  (0.05) | 1427 (0.02) | 4070 (0.03) |
| I47 | Paroxysmal tachycardia | 1335  (0.02) | 1811 (0.02) | 3146 (0.02) |

**Table S2.** Top 10 health services & drugs consumed 30-day prior index hospitalisations

| Health services | Frequency (rate) | | | Medications | | Frequency (rate) | | | | |  | |
| --- | --- | --- | --- | --- | --- | --- | --- | --- | --- | --- | --- | --- |
|  | Rec | Inc | All |  |  | Rec | | Inc | All | | |  |
| GP attendance | 42468 (0.75) | 58102 (0.77) | 100570 (0.76) | Clopidogrel | 7921 (0.14) | | 2679 (0.04) | | | 10600 (0.08) | |  |
| Specialist attendance | 26629 (0.47) | 36559 (0.48) | 63188 (0.48) | Perindopril | 7055 (0.12) | | 3647 (0.05) | | | 10702 (0.08) | |  |
| Consultant physician attendance | 27540 (0.49) | 33394 (0.44) | 60934 (0.46) | Furosemide | 5803 (0.1) | | 1952 (0.03) | | | 7755 (0.06) | |  |
| Non-referral attendance | 18193 (0.32) | 32550 (0.43) | 50743 (0.38) | Warfarin | 4845 (0.09) | | 1731 (0.02) | | | 6576 (0.05) | |  |
| Anaesthetics | 23256 (0.41) | 25308 (0.33) | 48564 (0.37) | Atenolol | 4757 (0.08) | | 3781 (0.05) | | | 8538 (0.06) | |  |
| Ultrasound | 19907 (0.35) | 16697 (0.22) | 36604 (0.28) | Simvastatin | 4748 (0.08) | | 3349 (0.04) | | | 8097 (0.06) | |  |
| GP Management plan | 11765 (0.21) | 15165 (0.20) | 26930 (0.20) | Ramipril | 4684 (0.08) | | 2170 (0.03) | | | 6854 (0.05) | |  |
| Surgical operations | 10865 (0.19) | 8151 (0.11) | 19016 (0.14) | Isosorbide mononitrate | 4526 (0.08) | | 1333 (0.02) | | | 5859 (0.04) | |  |
| Diagnosis radiology | 8925 (0.16) | 11746 (0.15) | 20671 (0.16) | Rosuvastatin | 4338 (0.08) | | 4046 (0.05) | | | 8384 (0.06) | |  |
| Urgent attendance | 6496 (0.11) | 4768 (0.06) | 11264 (0.09) | Metoprolol | 4205 (0.07) | | 1530 (0.02) | | | 5735 (0.04) | |  |

Rec=recurrent episodes, Inc=incidence episodes; All=all index hospitalisations; χ^2^ test reveal p<0.001 for all pairs.
